# Primary omental torsion mimicking acute appendicitis in a child: a case report

**DOI:** 10.1093/jscr/rjag446

**Published:** 2026-06-10

**Authors:** Salam Melhem, Hadeel I Bouzia, Nour H Moosa

**Affiliations:** Head of Department of Surgery, Qalqilya Hospital (UNRWA), Nablus Road, Qalqilya, West Bank, Palestine; Faculty of Medicine, Palestine Polytechnic University, Wad Alharya, Hebron, West Bank, Palestine; Faculty of Medicine, Palestine Polytechnic University, Wad Alharya, Hebron, West Bank, Palestine

**Keywords:** omental torsion, acute abdomen, appendicitis, laparoscopy

## Abstract

Omental torsion is a rare cause of acute abdomen and an uncommon entity in the pediatric population, often mimicking more common surgical conditions such as acute appendicitis. Preoperative diagnosis remains challenging due to nonspecific clinical and radiological findings. We report the case of an 8-year-old boy who presented with acute right-sided abdominal pain and ultrasound findings initially suggestive of early acute appendicitis, which later proved to be misleading. Diagnostic laparoscopy revealed a hemorrhagic, infarcted omental mass near the hepatic flexure, along with a mildly congested but non-inflamed appendix. Laparoscopic excision of the omental mass and appendectomy were performed. Histopathology confirmed primary omental torsion, without appendicitis. The postoperative course was uneventful. This case highlights the diagnostic difficulty of pediatric omental torsion and supports the role of laparoscopy as an effective diagnostic and therapeutic tool in children presenting with features of an acute abdomen.

## Introduction

Omental torsion is a rare cause of acute abdomen, often mimicking acute appendicitis [1]. The greater omentum protects by isolating inflammation but, due to its mobility—especially on the right side—is prone to vascular torsion [2]. It is classified as primary, occurring without an identifiable cause, or secondary, associated with intra-abdominal conditions such as adhesions, tumors, inflammation, or hernias [3]. Primary (unipolar) torsion is linked to obesity, abnormal omental anatomy, and vascular variation [4].

Although omental torsion predominantly affects adults, it is extremely rare in children, with an incidence of 0.024%–0.1% in pediatric appendicitis surgeries [5]. Clinical presentation is nonspecific, typically right-sided abdominal pain, with minimal gastrointestinal symptoms [2]. Preoperative diagnosis remains difficult despite imaging advances, and most cases are confirmed intraoperatively [5].

We report a pediatric case of primary omental torsion mimicking acute appendicitis, emphasizing diagnostic challenges and the role of laparoscopy.

## Case report

An 8-year-old boy (35 kg, 128 cm; BMI approximately 21.4, above the 95th percentile for his age and sex), with no significant past medical history, presented with a one-day history of progressively worsening right-sided abdominal pain. He appeared ill but was hemodynamically stable. Vital signs, including temperature, pulse, blood pressure, and oxygen saturation, were within normal limits. Abdominal examination was consistent with an acute abdomen, demonstrating guarding and rebound tenderness, more pronounced over the right iliac fossa, right flank, and right lumbar region. No abdominal distension was noted.

Laboratory investigations revealed mild leukocytosis (WBC 10.76 × 10^3^/μL), an elevated platelet count (482 × 10^3^/μL), and a normal hemoglobin level (13.1 g/dL). Renal function tests, liver enzymes, electrolytes, coagulation profile, and urinalysis were all within normal limits.

An initial abdominal ultrasound showed slightly echogenic fat in the right iliac fossa with a normal appendix (<6 mm) and incidental multiple small gallstones. A repeat ultrasound performed the following day demonstrated a partially visualized, non-compressible appendix measuring 6.5 mm, with surrounding echogenic fat planes, minimal free fluid, and prominent mesenteric lymph nodes, suggestive of early acute appendicitis; clinical correlation was advised. The patient received IV fluids, cefuroxime, metronidazole, famotidine, and paracetamol preoperatively.

**Figure 1:**
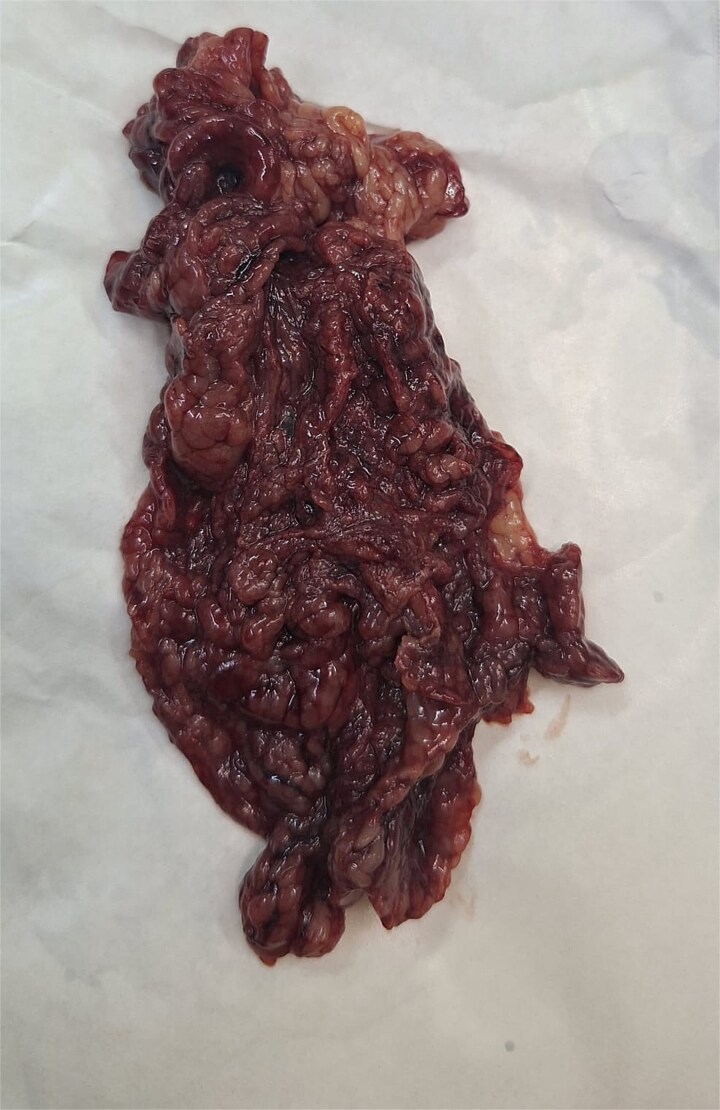
Laparoscopic view of the excised omental mass measuring approximately 10 × 5 cm, demonstrating a hemorrhagic and infarcted segment consistent with omental torsion.

Diagnostic laparoscopy revealed a mildly congested appendix without evidence of acute inflammation and a hemorrhagic, infarcted omental mass in the right upper quadrant near the hepatic flexure. Approximately 100 mL of serosanguinous fluid was aspirated. Laparoscopic appendectomy and excision of the omental mass (Fig. 1) were performed with minimal blood loss. The specimens were retrieved using an endobag and sent for histopathological examination.

Histopathological examination of the omental mass confirmed torsion, demonstrating lobules of mature adipose tissue with hemorrhage and reactive inflammation. The appendix showed a fecolith and lymphoid hyperplasia, with no evidence of acute appendicitis or malignancy.

Recovery was uneventful, and the patient was discharged on postoperative Day 1 with oral antibiotics and analgesics. Follow-up during the first week was unremarkable.

## Discussion

The spleen, gallbladder, omentum, fallopian tube, epiploic appendages, and epididymis/testicular appendix are uncommon but important causes of acute abdomen that may mimic more frequent surgical conditions [1]. The greater omentum normally limits inflammation but, due to its mobility—particularly on the right side—is prone to torsion along its vascular axis [2].

Omental torsion is classified as primary or secondary based on its underlying mechanism. Primary torsion, also referred to as unipolar torsion, occurs when the distal end of the omentum remains free [3], although its exact cause remains unclear. Several risk factors have been identified, including vascular anomalies, anatomical variations of the omentum, and obesity [4]. In this case, the patient’s obesity likely contributed to the condition by increasing omental fat, which can enhance mobility and risk of torsion. This risk may be further increased by triggers such as sudden movements or rises in intra-abdominal pressure [4]. In contrast, secondary torsion is typically bipolar and associated with underlying intra-abdominal pathology, including adhesions, hernias, inflammatory conditions, or prior surgery [5].

A comprehensive literature review from 1986 to 2024 identified 236 articles reporting 479 cases of omental infarction and torsion. The condition demonstrates a clear male predominance (approximately 2:1) and is significantly more common in adults than in children [6]. The reported incidence in the pediatric population ranges from 0.024% to 0.1% among children undergoing surgery for suspected acute appendicitis [5].

Clinically, right lower quadrant pain is the most common presentation in children, often leading to suspicion of appendicitis [2]. Differential diagnoses include mesenteric lymphadenitis, intussusception, ascariasis, and Henoch–Schönlein purpura; omental torsion is rarely considered [5]. A key feature is the absence of prominent gastrointestinal symptoms such as vomiting or bowel disturbance [7], In our case, the patient presented primarily with localized pain without significant gastrointestinal symptoms, consistent with previously reported cases [5, 7].

Laboratory findings are typically nonspecific, with mild leukocytosis being the most commonly reported abnormality [2]. Similarly, our patient showed only mild leukocytosis with otherwise normal labs, highlighting the limited diagnostic value of laboratory findings and the importance of clinical assessment alongside imaging and, when needed, surgical exploration. Despite advances in imaging, most cases are diagnosed intraoperatively due to nonspecific findings [5].

Ultrasonography may demonstrate a non-compressible hyperechoic mass; however, its primary role is often to exclude more common conditions such as appendicitis [8]. In our case, ultrasonography findings were inconsistent and potentially misleading, with initial imaging showing a normal appendix and subsequent imaging suggesting early appendicitis. This variability highlights the operator-dependent nature and limited specificity of ultrasonography in such cases. Computed tomography (CT) is more sensitive and may reveal characteristic features such as the ‘whirl sign’ [8]. CT imaging was not done due to limited resources and the patient’s worsening condition, Consequently, diagnostic laparoscopy was performed, serving both diagnostic and therapeutic purposes.

Surgical intervention remains the most common management approach, particularly in cases with diagnostic uncertainty [6]. Laparoscopy typically reveals a twisted, infarcted omental segment with serosanguinous fluid and a normal or mildly inflamed appendix, findings that were consistent with our intraoperative observations [4–6].

Appendectomy was performed despite the absence of acute inflammation due to preoperative suspicion and imaging findings. This approach is commonly used to avoid future diagnostic confusion and reoperation and is supported in similar reports [5, 9, 10].

This case highlights the importance of considering omental torsion in children with right-sided abdominal pain, minimal gastrointestinal symptoms, inconclusive imaging, and disproportionate clinical findings. Early laparoscopy is essential for diagnosis and management.
